# Changing paradigms in management of inflammatory bowel disease

**DOI:** 10.1002/ueg2.12347

**Published:** 2022-12-05

**Authors:** Ren Mao, Fernando Magro

**Affiliations:** ^1^ Department of Gastroenterology The First Affiliated Hospital of Sun Yat‐sen University Guangzhou China; ^2^ Department of Gastroenterology São João Hospital Center Porto Portugal; ^3^ CINTESIS ‐ Center for Health Technology and Services Research Porto Portugal

In the last decade, the prevalence of inflammatory bowel disease (IBD) has increased, both in developed and developing regions, with a marked global burden associated with disease management.^1^ Our knowledge of IBD pathogenesis, diagnosis, monitoring, and treatment strategy has changed substantially over the last years. To collect the latest evidence and make it available to healthcare professionals, we, hereby, present an IBD special issue focused on the most recent monitoring and treatment innovations.

## EPIDEMIOLOGY AND RISK FACTORS OF IBD

Even though the etiology of IBD remains unknown, it has been hypothesized that genetic heterogeneity, environmental factors, microbiome dysbiosis, and immune disorders act together to promote the development of the disease.^1^ To further understand the complex pathogenesis of IBD, it is crucial to focus on the recent changing trends of its epidemiology, especially in newly industrialized regions such as Asian countries. Aniwan et al.^2^ discussed the increased incidence and prevalence of IBD, in the last decades, in both Asian countries and Asian immigrants in western countries. The discussion of this data shall consider that this population exhibits a unique disease phenotype, with a high rate of perianal disease in Crohn's disease (CD). These changing patterns provide relevant insights into our understanding of IBD risk factors such as environment, lifestyle, and health‐related factors. In this context, Singh et al.^3^ reported that early life exposures, lifestyle and hygiene, vaccinations, exposure to drugs, and gastrointestinal pathogens may increase the risk of developing IBD. In recent years, the link between microbiome and IBD pathogenesis, and the application of microbiome‐based biomarkers in IBD precision medicine have been approached with great interest. In line with this, Zheng et al.^4^ summarized the current evidence regarding the impact of microbiota on IBD pathogenesis and discussed the latest literature on the utilization of potential microbial biomarkers in predicting disease risk, disease progression, and responses to therapeutics. Understanding the impact of the above‐mentioned environmental factors and microbiome on IBD risk is vital for the development of IBD burden‐attenuating strategies, such as tertiary prevention and primary prevention.^5^

## DISEASE EVALUATION AND MONITORING

Endoscopy is the most widely used tool to evaluate IBD activity and assess treatment efficacy. In fact, in the last decade, IBD treatment targets have evolved towards objective measures of disease activity, which can be assessed by endoscopic techniques.^6^ Nardone et al.^7^ provided an overview of the endoscopic tools to measure mucosal inflammation and healing in IBD, with a particular focus on advanced techniques such as endocytoscope and confocal laser endomicroscopy. In addition, cross‐sectional imaging modalities are being used as complementary tools to endoscopy, enabling the assessment of transmural lesions and extraluminal complications in IBD. In this field, Wang et al.^8^ provided an update on cross‐sectional imaging advances including magnetic resonance enterography (MRE) and intestinal ultrasound (IUS) for IBD management, including extra‐luminal lesion detection, evaluation of complications, therapeutic response assessment, and outcome prediction. In addition, Sleiman et al.^9^ emphasized the novel cross‐sectional imaging parameters used for the characterization of stricturing CD, including differentiation between inflammatory, muscular, and fibrotic stricture components.

It is expected that IBD management will soon benefit from the advances in the field of artificial intelligence (AI), which has a powerful ability in information processing and has already revolutionized many areas of medicine.^10^ The modalities and impact of the implementation of AI in endoscopy and cross‐sectional imaging modalities on the clinical management of IBD have been already discussed in several invited reviews.^7^, ^8^, ^9^

## MANAGEMENT OF COMPLICATIONS

The management of IBD‐associated complications, including intestinal stricture, penetrating disease, and colorectal cancer (CRC), is challenging. Patients with IBD and longstanding colonic inflammation are at an increased risk of developing CRC.^11^ However, current surveillance programs present several limitations, including low levels of patient enrollment, missed lesions (resulting in interval cancers), and uncertainties in the management of dysplasia.^11^ In a 2022 review, AI Bakir et al.^12^ discussed the histopathological diagnosis of IBD‐associated dysplasia and described the techniques to maximize dysplasia detection. Moreover, the review proposed a practical and standardized multi‐disciplinary approach for the management of patients with dysplasia.

Regarding treatment, Haas et al.^13^ summarized new data regarding the efficacy and safety of anti‐TNF drugs in the management of intra‐abdominal abscesses complicating CD. This review discussed the “no surgery” strategy, which starts with antibiotic therapy combined with radiologic drainage, followed by anti‐TNF therapy, avoiding delayed surgery.^13^ Future studies are warranted to clarify the type of patient and drug, including vedolizumab and ustekinumab, to be considered in this setting.

## SEQUENCING THERAPY AND LANDSCAPE OF NEW TREATMENT TARGETS

Creeping fat (CF), the wrapping of mesenteric fat around the bowel wall, is a hallmark of CD. Increasing evidence suggests a previously unrecognized role of CF in both intestinal inflammation and clinical complications.^14^ In this context, Yi et al.^15^ reviewed the molecular crosstalk between CF and intestinal inflammation, with focus on bacteria translocation and CF formation. Targeting the key mechanism of CF formation may provide relevant data for the development of a novel therapeutic strategy to improve CD outcomes.

With the emergence of new treatment options, a variety of factors shall be considered while selecting the most adequate therapeutic approach. A review by Garcia et al.^16^ focused on the activity and severity of the disease, comorbid illnesses, disease phase, and accessibility and affordability. To provide a landscape of new drugs in IBD, Vieujean et al. conducted an exhaustive search using ClinicalTrials.gov.^17^ The authors summarized and discussed the molecules which are currently being evaluated in phase 3 clinical trials and briefly described drugs under evaluation in phase 1 and 2 clinical trials.

In summary, this special issue covers a comprehensive review of the changing paradigms in the context of IBD management. The information herein provided will be instrumental in inspiring our healthcare professionals to improve the quality of care of IBD patients towards more personalized and appropriate treatment strategies.
